# The role of percutaneous transhepatic biliary biopsy in the diagnosis of patients with obstructive jaundice: an initial experience

**DOI:** 10.1590/0100-3984.2018.0073

**Published:** 2019

**Authors:** Tiago Kojun Tibana, Renata Motta Grubert, Vinicius Adami Vayego Fornazari, Fábio Colagrossi Paes Barbosa, Bernardo Bacelar, Amauri Ferreira Oliveira, Edson Marchiori, Thiago Franchi Nunes

**Affiliations:** 1 Hospital Universitário Maria Aparecida Pedrossian da Universidade Federal de Mato Grosso do Sul (HUMAP-UFMS), Campo Grande, MS, Brazil.; 2 Escola Paulista de Medicina da Universidade Federal de São Paulo (EPM-Unifesp), São Paulo, SP, Brazil.; 3 Laboratório Scapulatempo, Campo Grande, MS, Brazil.; 4 Hospital Regional de Mato Grosso do Sul, Campo Grande, MS, Brazil.; 5 Universidade Federal do Rio de Janeiro (UFRJ), Rio de Janeiro, RJ, Brazil.

**Keywords:** Jaundice, obstructive, Biliary tract, Biopsy/methods, Biopsy, needle/methods, Cholangiography, Icterícia obstrutiva, Trato biliar, Biópsia/métodos, Biópsia por agulha/métodos, Colangiografia

## Abstract

**Objective:**

To evaluate the accuracy of percutaneous transhepatic biliary biopsy (PTBB) in patients with suspected biliary obstruction.

**Materials and methods:**

This was a retrospective analysis of 18 patients with obstructive jaundice who underwent PTBB. In each patient, three to ten fragments were collected from the lesion. The final diagnosis was confirmed in the pathology report. We also reviewed analyses of the results of laboratory tests performed before the procedure, as well as the Bismuth classification, clinical outcome, complications occurring during the procedure, access route, and materials used.

**Results:**

Technical success was achieved in 100% of the PTBB procedures. Among the 18 patients clinically diagnosed with bile duct stenosis, the pathological analysis confirmed that diagnosis in 17. In one case, the pathological findings were considered false-negative. The predominant tumor was cholangiocarcinoma (seen in 50% of the cases). Sixteen of the procedures (88.9%) were performed without complications. Transient hemobilia occurred in one case, and cholangitis occurred in another.

**Conclusion:**

PTBB is a safe, viable, simple technique with a high rate of true-positive results for the definitive diagnosis of obstructive jaundice.

## Abstract (pt)

**Objetivo:**

Avaliar a precisão diagnóstica da colangiobiópsia trans-hepática percutânea (CBTP) em pacientes com suspeita de obstrução biliar.

**Materiais e Métodos:**

Análise retrospectiva de 18 pacientes apresentando icterícia obstrutiva foram submetidos a CBTP. Em cada paciente, 3 a 10 fragmentos foram coletados da lesão. O diagnóstico final foi confirmado por relatório anatomopatológico. Adicionalmente, foram registrados a análise laboratorial antes do procedimento, a classificação de Bismuth, o desfecho clínico, as intercorrências durante o procedimento, a via de acesso e os materiais utilizados.

**Resultados:**

A CBTP apresentou sucesso técnico em 100% dos casos. Dos 18 pacientes clinicamente diagnosticados com estenose biliar, 17 receberam diagnóstico patológico positivo. Em um caso os achados patológicos foram considerados falso-negativos. O colangiocarcinoma foi a neoplasia predominante (50%). Dezesseis (88,9%) procedimentos foram realizados sem intercorrências. Hemobilia transitória ocorreu em um caso e colangite em outro caso isolado.

**Conclusão:**

A CBTP é uma técnica segura, viável e simples, com alta taxa de verdadeiro-positivos para o diagnóstico definitivo de causas de icterícia obstrutiva.

## INTRODUCTION

Major advances in the diagnosis of biliary obstruction have been made in recent decades. The site of obstruction in the biliary tract can be identified quickly and accurately through the use of imaging modalities such as ultrasound, computed tomography (CT), and magnetic resonance cholangiopancreatography (MRCP), all of which are noninvasive^(^^1^^,^^2^^)^. However, tumors that affect the bile duct are often too small to be seen or to show specific image findings. In addition, malignant obstructions cannot be easily distinguished from benign obstructions^(^^1^^,^^3^^)^. Therefore, histological confirmation is often necessary in order to make the correct diagnosis, informing decisions regarding the treatment as well as the prognosis^(^^1^^)^. Interventional imaging techniques are gaining increasing prominence in the medical literature^(^^4^^-^^9^^)^.

Percutaneous transhepatic biliary drainage (PTBD) is a well-established procedure, performed using interventional radiology in patients with obstructive jaundice. It provides access to the bile duct for various biopsy instruments. Biopsies using PTBD access became a popular method for the diagnosis of biliary tumors since it was first described, in 1980^(^^10^^)^. More recent studies have suggested that a histological diagnosis by forceps biopsy was more successful than either bile cytology or fine-needle aspiration, having a reported sensitivity of 78-93%^(^^11^^-^^14^^)^.

The objective of this study was to evaluate the diagnostic accuracy of percutaneous transhepatic biliary biopsy (PTBB). To that end, we evaluated 18 patients with suspected biliary obstruction.

## MATERIALS AND METHODS

This study was approved by the local institutional review board. Because of the retrospective nature of the study, the requirement for informed consent was waived. 

We retrospectively evaluated 18 consecutive patients (10 males and 8 females; 55-75 years of age; mean age, 64.8 years) who presented with obstructive jaundice and underwent percutaneous transluminal biopsy of the bile duct with a 50-cm flexible 5.4-F biopsy forceps (Cordis; Miami, FL, USA) between January 2017 and March 2018. We excluded five patients for whom no pathology or imaging reports were available.

Before the PTBB procedure, the patients were submitted to either CT (in 16.6%) or MRCP (in 83.4%). In the retrospective analysis of the imaging examinations, three radiology residents applied the Bismuth classification^(^^15^^)^, and the classifications were reviewed by two interventional radiologists experienced in abdominal imaging. Disagreements were resolved by consensus. The procedures were performed by an interventional radiologist with ten years of experience in the treatment of the biliary tract, by means of PTBB during or after the bile duct decompression procedure. The histopathological analysis was performed by a pathologist with eight years of experience at a national referral hospital for treatment of the biliary tract. All data were tabulated and analyzed in a Microsoft Excel spreadsheet.

### Patient selection

Suspicion of a malignant obstruction was the main indication for biopsy. The medical records, surgical reports, histopathological reports, and diagnostic imaging reports of each patient were reviewed retrospectively. The following data were recorded: age; gender; technical success of the biopsy procedure; biopsy access route (left or the right); the lesion site; length of the stenosis; complications and outcome; number of fragments; procedures previously performed; and materials used in the PTBB. The patients were followed on an outpatient basis and were reassessed 30 days after hospital discharge. 

### Clinical and biochemical characteristics

The clinical characteristics included varying degrees of jaundice evidenced in the mucous membranes and sclera, dark urine, epigastric pain, fatigue, abdominal distension, lack of appetite, and, in some patients, cutaneous pruritus and acholic stools. Laboratory tests showed liver damage and increased levels of total and direct bilirubin.

### Final diagnosis

All patients received a pathological diagnosis obtained by PTBB. When the pathological diagnosis and clinical diagnosis were consistent, the pathological findings were considered positive. If no tumor was found in the pathology but imaging exams and clinical evidence supported the diagnosis of a tumor, the pathology findings were considered negative.

### Obstructive jaundice and level of obstruction (Bismuth type)

To determine the level of obstruction based on imaging exams, we used the Bismuth classification^(^^15^^)^, which is based on the level of the lesion (the point above and below which healthy biliary mucosa is found) in relation to the confluence of the hepatic ducts. The classification divides lesions into five types^(^^15^^)^: type I - distal stenosis of the common bile duct and the stump of the common hepatic duct being > 2 cm in length; type II - proximal stenosis of the common bile duct and the stump of the common hepatic duct being < 2 cm in length; type III - hilar stenosis with preservation of the confluence of hepatic ducts; type IV - hilar stenosis without preservation of the confluence of hepatic ducts and with a loss of communication between the branches of the right and left hepatic ducts; type V - stenosis of the aberrant right hepatic duct, with or without concomitant stenosis of the common hepatic duct.

### The PTBB technique

For conscious sedation, midazolam and fentanyl were administered intravenously before the procedure. All biopsies were performed under local anesthesia (2% lidocaine at the puncture site). 

First, the bile duct (left or right) was punctured, the puncture site being selected on the basis of previous imaging exams. That is followed by cholangiography with a right anterior oblique projection, the objective of which is to visualize the point of obstruction, as well as the morphology and length of the stenosis. After the transposition the stenosis, an angled introducer sheath was implanted in the region to be biopsied. If an 8F sheath was chosen, a 0.014-inch guidewire was used, whereas a 0.035-inch guidewire was used if a 9F sheath was chosen. Using endoscopic through-the-needle forceps, we collected fragments of various portions of the lesion from the region selected in the pre-planning. At the end of the procedure, a biliary drain was inserted with its distal end either in the duodenum or external, the latter for cases in which there was technical difficulty in overcoming the stenosis. 

### Definition of technical success

The biopsy procedure was considered technically successful when the site of the lesion was accessed, tissue samples were acquired, and a conclusive histopathological diagnosis was obtained. The sampling was considered successful if the pathologist received enough material to provide a diagnosis. 

## RESULTS

**Figure 1:**
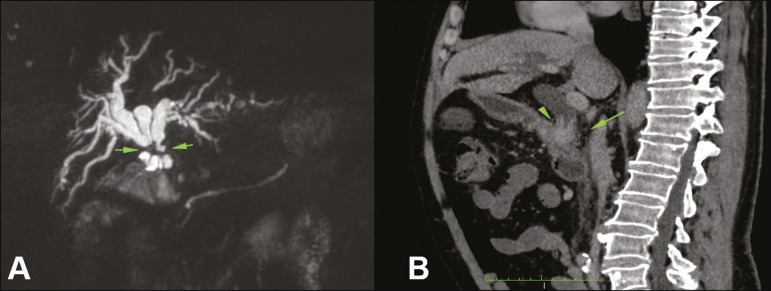
**A:** MRCP of patient 7, showing Bismuth type IV stenosis, 2.5 cm in length. **B:** Contrast-enhanced sagittal CT scan of the abdomen of patient 18, showing Bismuth type I stenosis, 3 cm in length.

The sample comprised 18 patients. As can be seen in Table 1, total bilirubin levels ranged from 10.5 mg/dL to 29.5 mg/dL (mean, 17.16 mg/dL) and direct bilirubin levels ranged from 9.6 mg/dL to 25.9 mg/dL (mean, 15.75 mg/dL). The mean length of the lesion was 1.8 cm (range, 1.0-3.5 cm). Eight lesions (44.4%) were classified as Bismuth type IV (Figure 1), four (22.2%) were classified as Bismuth type III, four (22.2%) were classified as Bismuth type I (Figure 1), and two (11.1 %) were classified as Bismuth type II. Prior to the PTBB, 9 (50.0%) of the patients had undergone endoscopic retrograde cholangiopancreatography (ERCP) at least once, two (11.1%) had undergone ERCP and biliary prosthesis insertion, and seven (38.9%) had not undergone any prior intervention (Table 1).

**Table 1:** Characteristics of the patients and lesions, together with the results of the biochemical analyses and the types of procedures previously performed.

Patient	Age (years)	Gender	Size of the lesion (cm)	Bismuth type	Total bilirubin (mg/dL)	Direct bilirubin (mg/dL)	Previous procedure(s)
1	75	Female	1.5	III	20.4	17.5	ERCP
2	60	Female	1.0	III	10.5	9.6	ERCP
3	59	Male	2.0	III	29.5	25.9	ERCP
4	65	Male	1.3	IV	15.4	13.6	ERCP
5	71	Male	2.5	IV	19.3	17.9	ERCP
6	67	Male	2.0	I	18.8	16.2	ERCP + biliary stent
7	76	Female	2.5	IV	20.1	18.3	ERCP
8	60	Male	3.0	IV	12.0	11.2	-
9	55	Female	3.5	IV	22.1	21.0	ERCP + biliary stent
10	64	Male	2.5	III	18.4	16.5	-
11	72	Female	1.6	IV	15.4	14.2	-
12	66	Female	2.3	II	13.8	12.1	-
13	70	Male	1.9	IV	15.1	14.1	ERCP
14	59	Male	2.1	IV	16.7	15.2	ERCP
15	65	Female	1.5	I	18.3	17.1	ERCP
16	61	Female	2.0	II	14.8	13.5	-
17	59	Male	2.5	I	12.5	11.5	-
18	68	Male	3.0	I	15.8	13.7	-

**Figure 2:**
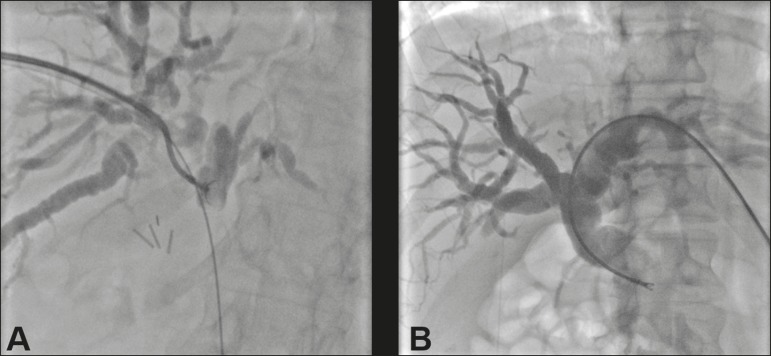
**A:** Puncture of the right bile duct and biliary biopsy with through-the-needle forceps in a patient with Bismuth type II stenosis. The pathology study revealed cholangiocarcinoma. **B**: Puncture of the left bile duct in a patient with Bismuth type I stenosis. The pathology study revealed liver metastasis of a colorectal carcinoma.

The procedures were technically successful, and the pathology analysis demonstrated that PTBB had a sensitivity of 94.4% for the diagnosis biliary obstruction. The biopsies were performed with a right-sided approach in 11 patients and a left-sided approach in 7 patients (Figure 2). The mean number of fragments was 5.38 (range, 3-10), exactly five fragments having been collected in 13 patients (72.2%). In 16 (88.9%) cases there were no complications before or during the procedure. As shown in Table 2, the postprocedural complications were hemobilia (n = 1) and cholangitis (n = 1). Table 2 also shows the details of the procedure. We used 8F sheaths in 14 cases (77.8%), 9F sheaths in 2 cases (11.1%), and 10F sheaths in 2 cases (11.1%). In two cases (11.1%), external drain placement was not required; a 10F drain was placed in nine patients (50.0%); and a 12F drain was placed in seven patients (38.9%). 

**Table 2:** PTBB analysis.

Patient	Access	Sheath size	Fragments	External drain	Pathology	Discharge	Complication
1	Right lobe	9F	6	10F	Cholangiocarcinoma	Postprocedure day 1	Hemobilia
2	Right lobe	8F	7	10F	Metastasis from an esophageal tumor	Postprocedure day 1	-
3	Left lobe	8F	5	12F	Metastasis from a colorectal carcinoma	Postprocedure day 1	-
4	Right lobe	9F	6	12F	Cholangiocarcinoma	Postprocedure day 2	-
5	Bilateral	8F	5	12F	Cholangiocarcinoma	Postprocedure day 3	-
6	Right lobe	8F	5	12F	Atypia	Postprocedure day 4	-
7	Bilateral	10F	5	10F	Cholangiocarcinoma	Postprocedure day 4	-
8	Left lobe	8F	5	10F	Cholangiocarcinoma	Postprocedure day 2	-
9	Left lobe	8F	3	10F	Absence of malignancy	Postprocedure day 4	-
10	Bilateral	8F	5	10F	Cholangiocarcinoma	Postprocedure day 4	Cholangitis
11	Right lobe	8F	5	10F	Cholangiocarcinoma	Postprocedure day 2	-
12	Right lobe	8F	5	10F	Metastasis from a breast tumor	Postprocedure day 1	-
13	Right lobe	8F	5	12F	Cholangiocarcinoma	Postprocedure day 2	-
14	Right lobe	8F	5	12F	Cholangiocarcinoma	Postprocedure day 3	-
15	Left lobe	8F	5	12F	Pancreatic adenocarcinoma	Postprocedure day 1	-
16	Right lobe	8F	5	-	Liver metastasis from a gastric tumor	Postprocedure day 1	-
17	Right lobe	8F	5	10F	Pancreatic adenocarcinoma	Postprocedure day 1	-
18	Right lobe	10F	10	-	Pancreatic adenocarcinoma	Postprocedure day 1	-

**Figure 3:**
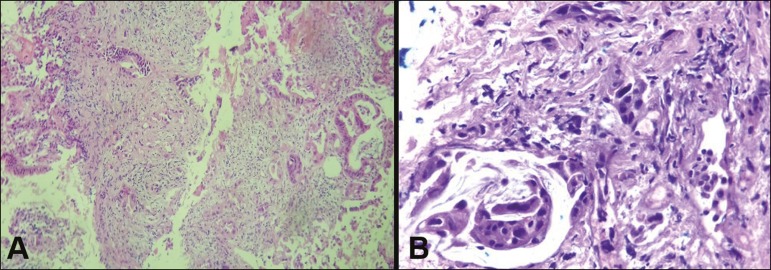
**A:** Adenocarcinoma composed of atypical cells, forming tubuloacinar and cribriform structures infiltrating connective tissue in the middle of the desmoplastic stroma. **B:** Tumor glands containing atypical cells with hyperchromatic nuclei, forming an acinar structure, and isolated cells infiltrating the connective tissue.

The final diagnosis was malignancy in 17 patients (94.4%). One single case resulted in a false negative (5.5%), probably due to the fact that the patient had previously been submitted to insertion of a biliary prosthesis. As shown in Table 2 and Figure 3, the pathology reports revealed cholangiocarcinoma in nine cases (50.0%), metastases to various sites (stomach, colon/rectum, and breast) in four cases (22.2%), pancreatic adenocarcinoma in three cases (16.7%), and nonspecific cellular atypia in one case (5.5%). On average, the patients were discharged 2.1 days (range, 1-4 days) after the PTBB. 

## DISCUSSION

A diagnosis of malignant neoplasm of the biliary tract is usually delayed, often preventing the start of proper treatment. It is difficult to obtain a pathological diagnosis, and the success rate of radical resection is low, leading to a poor prognosis^(^^16^^,^^17^^)^. The PTBB provides a pathological basis for the treatment of cancer and, at the same time, a treatment for obstructive disease^(^^17^^)^.

Currently, several transluminal techniques for the acquisition of biliary tumor tissue are performed by means of PTBD. Because most tumors of the bile duct originate from the ductal epithelium, the tissue obtained from the bile duct of an abnormal segment seems to be the most suitable for pathological examination. Although the collection of bile for cytological examination is a simple technique, it is rarely used because it produces unsatisfactory results^(^^18^^)^. Sampling by brush cytology during PTBD or ERCP has been shown to be safe and effective, although several reports have shown that it has relatively low sensitivity and that the specimens collected can be superficial or insufficient^(^^11^^,^^19^^,^^20^^)^. Despite the fact that the specificity of the method is considered high for detection of malignancy^(^^21^^)^, it hampers the subtyping of the neoplasia and the identification of the primary site. 

In an attempt to further increase the diagnostic value of the bile duct biopsy, a transluminal technique that incorporates the use of biopsy forceps has been developed; that technique has an estimated sensitivity of 78-93% and a specificity of 100% for the detection/exclusion of malignant biliary obstruction^(^^11^^-^^14^^)^.

In addition to saving time and reducing the number of procedures the patient must undergo, the use of the biliary drainage procedure to obtain tissue for histopathological analysis improves diagnostic accuracy for the pathologist and provides material for complementary studies (mainly immunohistochemical studies) in cases in which the morphology or clinical suspicion indicates metastatic disease. An approximate average of five fragments proved to be satisfactory in this study. It should be noted that highly heterogeneous lesions may require more extensive sampling and sampling from different areas, to avoid the (non-diagnostic) necrotic or fibrotic component of the tumor. The storage and fixation of the material must be done in the usual manner, in 10%, preferably buffered, formalin.

In keeping with their biological behavior, most metastatic biliary tumors or adjacent tumors that invade the bile duct are moderately differentiated carcinomas. The less differentiated the tumor, the greater the degree of malignancy and the higher the chances are of it invading adjacent structures and metastasizing^(^^22^^,^^23^^)^.

The results of a PTBB for a non-biliary carcinoma causing bile duct obstruction are influenced by the extent of the infiltration of the bile duct, and the rate of false-negative results can be high^(^^17^^)^. The infiltration of the bile duct varies among tumors at different sites and with different patterns of infiltration. For example, a hepatic or pancreatic carcinoma can impact the bile duct and cause obstruction, whereas carcinoma of the gallbladder can infiltrate the bile duct directly or via lymphatic metastasis and dissemination from the bile duct, and metastases from gastrointestinal tumors may spread to the porta hepatis or hepatoduodenal ligament, leading to lymph node enlargement and compression or infiltration of the bile duct^(^^17^^)^. If the wall of the duct is infiltrated, biopsy forceps can easily be used to collect pathological samples from the surface of the lesion, in which case the true-positive rate is high. Otherwise, the forceps must be passed through the mucosa to obtain a sample of the diseased tissue, in which case the true-positive rate is relatively low^(^^17^^)^.

The present study demonstrated that the PTBB has high (94.4%) sensitivity for diagnosing biliary obstruction, especially obstruction caused by malignancy. Several studies^(^^11^^,^^13^^,^^14^^,^^24^^)^ with relatively large patient samples have obtained results comparable to ours, reporting PTBB sensitivity values ranging from 78% to 94%. We observed a false-negative result in only one case, probably due to previous placement of a biliary prosthesis and balloon dilation. In such cases, because the bile duct is no longer narrowed, the terminus of the sheath no longer has a substrate and can slide to the distal part of the lesion, from which it is extremely difficult to collect a biopsy sample^(^^17^^)^. In the three cases in which a diagnosis of pancreatic cancer was made in the present study, the quantity of material obtained through PTBB was sufficient for diagnosis. Other reported transluminal biopsy techniques include the use of a Simpson atherectomy catheter^(^^25^^,^^26^^)^. One such technique was reported to have a sensitivity of 79%^(^^26^^)^, comparable to that obtained with PTBB. However, using an atherectomy catheter has certain disadvantages, mainly the difficulties involved in using a rigid instrument in the sharply angled biliary tract and the high cost of the device, as well as the risk of injury and hemorrhage of the biliary tract. One previous study showed that 11% of the patients evaluated experienced transitory but clinically relevant hemorrhage^(^^26^^)^.

In the present study, the rate of complications was low (11.1%), one patient showing postprocedural hemobilia and another showing postprocedural cholangitis. In theory, significant hemorrhage could occur as a complication of a PTBB when there is bile leakage or an adjacent vascular lesion^(^^27^^)^. However, such complications have not been reported in the literature and were not seen in our study. 

In one previous study, involving forceps biopsies of the bile duct guided by percutaneous transhepatic cholangioscopy, the biopsy materials were obtained only from the mucosa and superficial part of the fibromuscular layer of the duct, suggesting that a forceps biopsy is less useful for detecting extrinsic tumors or tumors in the deep part of the bile duct wall^(^^28^^)^. Our study showed that the sensitivity of forceps biopsy for the diagnosis of malignant tumors other than cholangiocarcinoma (33%) was similar to its sensitivity for the diagnosis of cholangiocarcinoma (50%). Another study^(^^27^^)^ reported a sensitivity of 100% for forceps biopsies, even in five patients with extrinsic malignancy, a result also seen in our study (in six patients). This discrepancy can be explained by differences in the depth of infiltration of the bile duct wall by the extrinsic malignancy.

In our experience with the PTBB technique, we noted that the procedure should be performed prior to balloon dilation, as discussed above, and that a blocked bile duct comprises a *nidus* surrounded by inflammatory edema^(^^29^^)^, with the lesion of limited size. Therefore, forceps biopsy should be used to obtain diseased tissue only from a limited region, otherwise a large quantity of inflamed tissue will be included. To improve the rate of true-positive results, the biopsy should be performed in multiple areas, with distinct characteristics, and in various directions. However, obtaining biopsy specimens from multiple points does not mean excessive sampling. Typically, only three to five samples are collected, because collecting a higher number of samples could increase the risk of complications.

Recent technological advances have resulted in the development of small-caliber, flexible fiberoptic endoscopes, which, together with knowledge of interventional radiology, have made cholangioscopy capable of allowing direct visualization of the site to be biopsied. These new tools represent promising alternatives in the attempt to facilitate the approach to complex cases, especially in the context of increased diagnostic accuracy of malignant obstructions of the biliary tract.

Our study has some limitations. First, it was a single-center, retrospective study, involving a small number of patients, and the high proportion of malignant diseases impaired the analysis of the negative predictive value of the method. In addition, all of the biopsy samples were analyzed by the same pathologist and there was no comparative analysis with another method such as brush cytology. We believe that further studies, preferably prospective, randomized, multicenter studies with significantly larger patient samples, are needed for validation of PTBB.

## CONCLUSION

From a technical point of view, PTBB is a simple procedure that is minimally invasive, with low complication rates and high diagnostic success rates in comparison with those of other techniques already described. Its use has broadened the scope of research on biliary disorders and, in clinical practice, it has proven to be a reliable, accurate method for the histopathological diagnosis of biliary neoplasms.
